# Effect of Expectation on Pain Processing: A Psychophysics and Functional MRI Analysis

**DOI:** 10.3389/fnins.2020.00006

**Published:** 2020-01-31

**Authors:** Luke A. Henderson, Flavia Di Pietro, Andrew M. Youssef, Sinjeong Lee, Shirley Tam, R. Akhter, Emily P. Mills, Greg M. Murray, Chris C. Peck, Paul M. Macey

**Affiliations:** ^1^Department of Anatomy and Histology, The University of Sydney, Sydney, NSW, Australia; ^2^School of Pharmacy and Biomedical Sciences, Curtin University, Perth, WA, Australia; ^3^Faculty of Dentistry, The University of Sydney, Sydney, NSW, Australia; ^4^UCLA School of Nursing and Brain Research Institute, University of California, Los Angeles, Los Angeles, CA, United States

**Keywords:** pain expectations, pain intensity, functional magnetic resonance imaging, pain modulation, somatosensory cortex

## Abstract

Pain is a complex phenomenon that is highly modifiable by expectation. Whilst the intensity of incoming noxious information plays a key role in the intensity of perceived pain, this intensity can be profoundly shaped by an individual’s expectations. Modern brain imaging investigations have begun to detail the brain regions responsible for placebo and nocebo related changes in pain, but less is known about the neural basis of stimulus-expectancy changes in pain processing. In this functional magnetic resonance imaging study, we administered two separate protocols of the same noxious thermal stimuli to 24 healthy subjects. However, different expectations were elicited by different explanations to subjects prior to each protocol. During one protocol, pain intensities were matched to expectation and in the other protocol they were not. Pain intensity was measured continuously via a manually operated computerized visual analogue scale. When individuals expected the stimulus intensity to remain constant, but in reality it was surreptitiously increased or decreased, pain intensity ratings were significantly lower than when expectation and pain intensities were matched. When the stimulus intensities did not match expectations, various areas in the brain such as the amygdala, anterior cingulate cortex (ACC), dorsolateral prefrontal cortex (dlPFC), and the midbrain periaqueductal gray matter (PAG) displayed significantly different patterns of activity compared to instances when stimulus intensity and pain expectations were matched. These results show that stimulus-expectancy manipulation of pain intensity alters activity in both higher brain and brainstem centers which are known to modulate pain under various conditions.

## Introduction

Our understanding of pain has evolved into a concept that combines sensation, emotion, cognition, and motivation. Not only is it is clear that pain can arise both with and without physical damage, one’s subjective sensory experience of pain can be profoundly shaped by interactions between expectations and the level of incoming sensory information (Wager et al., 2004; Atlas and Wager, 2012). Indeed, expectation of pain has been found to result in cortical activation patterns similar to those that modulate the sensory and affective aspects of pain (Porro et al., 2002). Studies have shown that altered expectations can change the pain experience, including the intensity and aversiveness of perceived pain (Lorenz et al., 2005; Atlas and Wager, 2012). While ability to predict pain has been shown to be an adaptive behavior in the healthy state (Ploghaus et al., 2003), it can cause avoidance and fear in chronic pain patients (Corbetta and Shulman, 2002). For this reason it is important to further our understanding of expectation, and the conflict that arises when expectations of pain are not matched with physical stimuli. The cognitive appraisal of pain during conflicting nociceptive inputs and expectation information has been explored, however, little is known about the underlying neural mechanisms involved in resolving such conflict.

A review by Atlas and Wager (2012) considered three main types of expectancy effects on pain processing (i) Placebo: in which an expectancy that a treatment will produce pain relief results in a pain reduction despite the treatment being inert; (ii) nocebo: in which an expectancy that a treatment will evoke an increase in pain results in a pain increase despite the treatment being inert; and (iii) stimulus expectancies: in which specific instructions or cues are used to induce expectations about the intensity of an upcoming stimulus. Whilst brain imaging studies have begun to unravel the neural sites responsible for placebo, and less often nocebo analgesia, few studies have explored stimulus-expectancy related changes in pain perception. The few studies that have explored stimulus expectancy have used auditory, visual, or innocuous somatosensory cues to alter expectation and perceived pain intensity and have reported the involvement of areas including the anterior cingulate cortex (ACC), dorsolateral prefrontal cortex (dlPFC), and insula (Atlas et al., 2010; Atlas and Wager, 2012; Lobanov et al., 2014). Whilst these studies did not specifically explore the brainstem, given the belief that most pain modulatory effects including placebo involve brainstem pain modulatory circuits such as the midbrain periaqueductal gray matter (PAG) (Koban et al., 2017), it is likely that the PAG is also involved in stimulus-expectancy related changes in pain intensity. The use of cues delivered immediately prior to a noxious stimulus to alter expectation likely alters attentional and emotional processing, in addition to areas related to processing pain intensity. Furthermore, such cues are inherently tied to either increases or alternatively to decreases in pain intensity expectation, a situation which may affect the manner in which the brain modulates noxious information.

**FIGURE 1:**
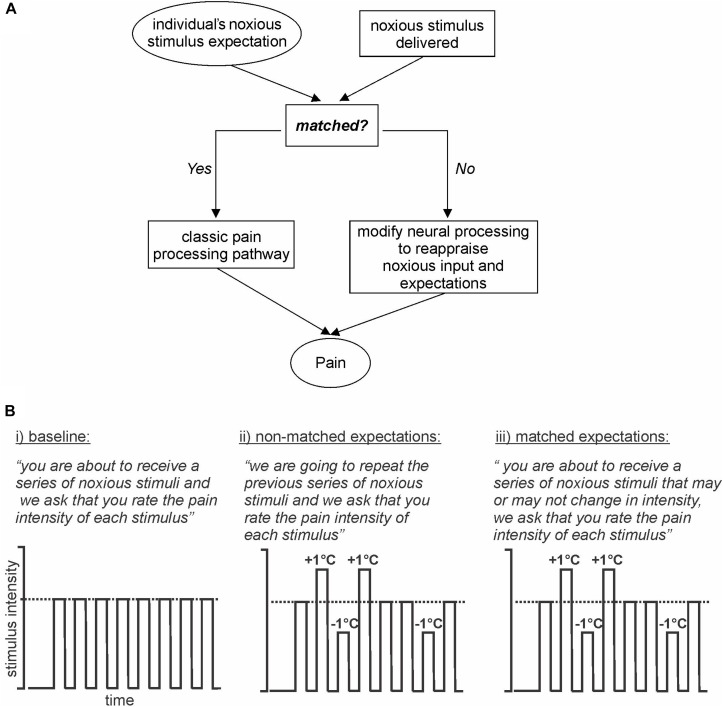
Model and Experimental design. **(A)** Model: basic model via which expectation of an incoming stimulus is either matched or not matched to an individual’s expectation. If the stimulus and expectations are not matched then the conflict is reappraised. **(B)** Experimental design: (i) baseline: subjects are expecting a series of noxious stimuli and that is what they receive; (ii) non-matched expectations: subjects expect a series of noxious stimuli of equal intensity, however, the intensities are varied up and down by 1°C; (iii) matched expectations: subjects expect a series of noxious stimuli of potentially varying intensities and they receive varied stimulus intensities, the same series as delivered in the previous series.

In this investigation, we aim to deliver a stimulus expectancy design in which expectation is not altered by the presentation of sensory cues immediately prior to noxious stimuli. Instead, we will manipulate expectation by presenting a series of noxious stimuli of identical intensities and setting the expectation that a second identical series will be delivered. However, during the second series, some stimulus intensities will be increased and others decreased. A third series will then be delivered where expectation and stimulus delivery are matched (Figure 1A). We hypothesize that when stimulus intensity is increased above expectation, the magnitude of perceived pain intensity will be lower than that evoked by a matched expectation of the same stimulus intensity. Secondly, we hypothesize that when expectation and stimulus intensities are not matched, altered brain activation will occur in areas of the dorsolateral prefrontal, insular, and cingulate cortices and also in brainstem regions known to modulate incoming noxious stimuli such as the PAG. Finally, we hypothesize that the limbic brain regions will influence the primary somatosensory cortex to modulate the intensity of perceived pain.

## Materials and Methods

### Subjects

Twenty-four pain-free healthy subjects (13 females, mean [±SEM] age: 22.0 ± 0.4 years) were recruited for the study. Informed written consent was obtained for all procedures, which were conducted under the approval of the local Institutional Human Research Ethics Committees and in accordance with the Declaration of Helsinki. The functional magnetic resonance imaging (fMRI) data sets are unavailable as we did not seek specific approval from the Institutional Human Research Ethics Committee to share the data.

### MRI Scans

Prior to entering the MRI scanner, a 3 × 3 cm MRI compatible Peltier-element thermode (Medoc Ltd., Ramat Yishai, Israel) was secured to the skin of the right side of the mouth. To determine a temperature that evoked moderate pain ratings in each individual, the thermode temperature was raised with a Thermal Sensory Analyser (TSA-II, Medoc) from a baseline temperature of 32°C to various high temperatures between 44 and 49°C, in 0.5°C intervals. High temperatures at a random level between 44°C and 49°C were applied every 15 s for periods of 10 s, and during each period the subject rated the pain intensity using a Computerised Visual Analogue Scale (CoVAS). The CoVAS is a 0–10 scale, with 0 = no pain and 10 = worse imaginable pain and subjects rate their pain continuously using a sliding toggle. Based on these recordings, in each subject the temperature that evoked a pain intensity of approximately 6 out of 10 was defined as the “middle” temperature.

Each subject was then positioned supine onto the MRI scanner bed and placed into a 3 Tesla MRI scanner (Achieva, Philips) and their head immobilized in a 32-channel head coil. Three 350 s fMRI series were collected, each consisting of 140 gradient-echo echo-planar image sets with Blood Oxygen Level Dependent (BOLD) contrast. Each image volume covered the entire brain, extending caudally to include the upper cervical spinal cord (38 axial slices, repetition time = 2500 ms, raw voxel size = 1.5 × 1.5 × 4.0 mm thick). The Medoc system was left in place, and was used to deliver heat stimuli during scanning. A different paradigm was then performed for each functional series:

1. *First fMRI scan (baseline: no expectation of pain magnitude, all stimuli same):* Subjects were instructed *“you are about to receive a series of noxious stimuli and we ask that you rate the pain intensity of each stimulus.”* Then, following a 30-volume baseline period, eight noxious thermal stimuli were delivered. These were all delivered at the same “middle” temperature determined during pre-testing. Each noxious stimulus was delivered for 15 s (including ramp up and down periods of 2.5 s each), followed by a 12-s period of baseline temperature (32°C). During each period of noxious stimulation, the subject was asked to rate the pain intensity continuously online using the CoVAS sliding toggle (Figure 1B, part i).
2. *Second fMRI scan (non-matched expectations: expecting same pain magnitude, varying stimuli delivered):* Subjects were instructed *“we are going to repeat the previous series of noxious stimuli and we ask that you rate the pain intensity of each stimulus.”* Subjects were therefore expecting the same eight identical temperature stimuli. However, unknown to the subject, the stimulus intensity was varied by increasing or decreasing the applied temperature by 1°C. That is, four stimuli were applied at the middle temperature, two stimuli at +1°C (“higher”), and two stimuli at −1°C (“lower”) (Figure 1B, part ii).
3. *Third fMRI scan (matched expectations: expecting varying pain magnitude, varying stimuli delivered):* Subjects were instructed *“you are about to receive a series of noxious stimuli that may or may not change in intensity, and we ask that you rate the pain intensity of each stimulus as it is delivered.”* Subjects were therefore expecting stimuli temperatures to vary. Indeed, we then varied the stimulus intensity to match the nature and order of the stimuli delivered during the second fMRI scan. That is, four stimuli were applied at the middle temperature, two stimuli at +1°C (higher), and two stimuli at −1°C (lower) (Figure 1B, part iii).

At the completion of the third fMRI scan, a T1-weighted anatomical image was collected (288 axial slices, repetition time = 5600 ms, raw voxel size = 0.87 × 0.87 × 0.87 mm). At the end of the MRI scanning session, each subject completed the Pain Catastrophizing Scale (Sullivan et al., 1995) and the Fear of Pain Questionnaire (McNeil and Rainwater, 1998). These are two of the most widely used measures of catastrophic thinking related to pain, and fear of pain, respectively.

### Pain Intensity Analysis

For each subject, the mean pain intensity rating during each noxious stimulus was determined for the baseline, non-matched expectations, and matched expectations fMRI scans. For the non-matched expectations and matched expectations scans, changes in pain intensity during the higher (+1°C) relative to the middle temperature, and during the lower (−1°C) relative to the middle temperature were calculated. Identical analyses were performed for the baseline scans even though the same temperature (middle) was presented during all eight stimuli. That is, if an individual received higher stimuli during presentations three and six then these stimuli during the baseline scan were analyzed as “higher” and vice versa. Therefore, higher versus middle and middle versus lower temperature differences were calculated for each subject and differences between these values were determined between all three fMRI scans (*p* < 0.05, paired *t*-tests, two-tailed).

### MRI Analysis

Using SPM12 (Friston et al., 1995) and custom Matlab code, fMRI images were realigned and the effect of movement on signal intensity was modeled and removed from images. Subjects whose movements in any linear direction were greater than 1 mm were removed from further analysis; this resulted in analysis of 20 subjects. The fMRI images from these 20 subjects were linear detrended to remove global signal intensity drift, and each subject’s fMRI image set was co-registered to their own T1-weighted anatomical image. The T1-weighted image set was then spatially normalized to the Montreal Neurological Institute (MNI) template and the normalization parameters applied to the fMRI images. The fMRI images were then spatially smoothed using a 6 mm full-width at half maximum (FWHM) Gaussian filter. In addition, using brainstem-specific isolation software (SUIT toolbox) (Diedrichsen, 2006), a mask of the brainstem was created individually for each subject for both the T1 and fMRI image sets. Using these masks, the brainstem of the T1 and fMRI image sets was isolated and then spatially normalized to a brainstem-specific template in MNI space and spatially smoothed using a 3 mm FWHM Gaussian filter.

Significant changes in signal intensity were determined using a repeated box-car model with the noxious stimulus periods given a value of “1” and baseline periods before and between noxious stimuli given a value of “0.” This repeated box-car model was then convolved with a canonical hemodynamic response function (using SPM12 functions) for analysis of signal intensity changes in both the wholebrain and brainstem-specific image sets. The closeness of fit between this repeated box-car model and signal intensity changes was determined for each voxel using a general linear model procedure. The resulting statistical maps were then used in second level random-effects analysis procedures to determine regions with differences in signal changes, within or between the three experimental fMRI scans. Firstly, for both the wholebrain and brainstem processed data, random-effects second level analyses were performed to determine significant signal intensity changes associated with each noxious stimulus presentation during the baseline session alone (*p* < 0.05, family-wise error rate corrected for multiple comparisons). Then, significant differences in signal intensity changes associated with each noxious stimulus period during the matched compared with the non-matched sessions were determined using a random-effects paired-group analysis. That is, the second comparison compared noxious stimulus evoked brain activation patterns during matched versus non-matched expectations. Whilst no voxels survived the relatively strict statistical threshold (*p* < 0.05, corrected), we followed the recommendation of Woo et al. (2014) and after a first-pass, voxel-based statistical threshold of *p* < 0.001 uncorrected and a minimum cluster size of five contiguous voxels, we performed a second pass cluster correction for multiple comparisons to reduce the likelihood of Type I errors. We only looked in areas pre-determined to be of interest, to further minimize Type I errors. Our regions of interest include the dlPFC, cingulate cortex, amygdala, and the midbrain PAG since it is well documented that these regions are involved in the processing and modulation of pain intensity. In the resulting significant clusters, the parameter estimate values indicating signal intensity change during each pain period were extracted and plotted for each individual subject. Furthermore, to ensure that movement related effects did not influence the results we calculated the standard deviation of movements in the *X*, *Y*, and *Z* planes and tilt, yaw, and roll rotations in each subject for each fMRI scan. We found that there were no significant differences in the variability of any of these six movement parameters between the non-matched and matched expectation scans (paired, *t*-test, all *p* > 0.05).

Finally, given our hypothesis that limbic brain regions may modulate pain by influencing higher brain regions involved in coding pain intensity, we performed a psychophysiological interactions analysis to determine noxious stimulus specific changes in connectivity using the wholebrain fMRI image sets. This type of analysis aims to determine the interaction between the noxious stimulus presentations and signal covariations in specific brain regions, i.e., the left and right dlPFC, left and right amygdala, and perigenual ACC. We determined significant signal covariations between these regions and each voxel in the contralateral primary somatosensory cortex (S1) during noxious stimuli relative to baseline periods during matched and non-matched expectation trials. The resulting connectivity maps were placed in second level random-effects analyses to determine significant differences in noxious stimulus related S1 connectivity during matched and non-matched trials. Given our hypothesis of noxious stimulus related connectivity differences within the orofacial region of S1, we applied small volume correction (*p* < 0.05) using the contralateral S1 region activated by noxious stimuli during the baseline trial to reduce the likelihood of Type I errors. For each of the resulting S1 significant clusters, the connectivity strengths were extracted, means (±SEM) calculated and connectivity values plotted for each individual subject during matched and non-matched trials.

## Results

### Pain Intensity Changes

**FIGURE 2:**
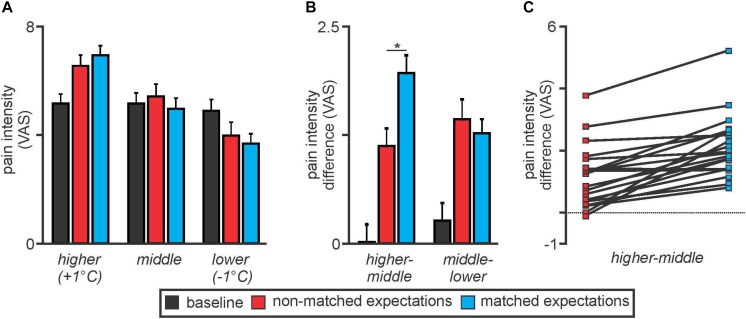
**(A)** Pain intensity ratings on an 11-point visual analogue scale (VAS) during middle, higher, and lower thermal intensities during the baseline (black), non-matched expectations (red), and matched expectations (blue) scans. Note that pain intensity ratings during the middle stimuli intensity presentations were stable at approximately 5 out of 10. Pain intensity ratings increased during the higher stimulus intensities and decreased during the lower stimulus intensities during both the non-matched and matched expectations scans. **(B)** Differences in pain intensity ratings during the higher versus middle and middle versus lower stimulus intensities. Note that when the increase in stimulus intensity was not expected, subjects rated the pain lower than when the increase was expected (higher–middle). A similar difference did not occur when the stimulus intensity was lower (middle–lower). *p < 0.05. **(C)** Individual subject pain intensity rating differences between higher and middle stimulus intensities. Note that almost all subjects rated the +1°C stimulus higher during matched compared with non-matched expectation trials.

Overall there were no significant differences in overall mean (±SEM) pain intensity ratings between the baseline (5.0 ± 0.4), non-matched expectation (5.2 ± 0.4), and matched expectation (5.0 ± 0.4) scans [baseline versus non-matched *T*(19) = 1.04, *p* = 0.31; baseline versus matched *T*(19) = 0.004, *p* = 0.98]. Furthermore, the mean pain intensity rating during the baseline scan was similar to the middle thermal stimulus temperature evoked pain intensity ratings during the non-matched and matched expectations scans [*baseline* 5.0 ± 0.4; *non-matched* 5.3 ± 0.5; *matched* 4.8 ± 0.4, baseline versus non-matched *T*(19) = 1.35, *p* = 0.19; baseline versus matched *T*(19) = 0.99, *p* = 0.34] (Figure 2A). During the higher stimulus intensity presentations, pain intensity ratings were higher during both the non-matched and matched expectation scans (*non-matched* 6.5 ± 0.4; *matched* 6.9 ± 0.3) and during the lower stimulus intensity presentations, pain intensity ratings were lower during both the non-matched and matched expectation scans (*non-matched* 4.0 ± 0.4; *matched* 3.7 ± 0.4).

Analysis of differences in pain intensity ratings during the higher compared with the middle stimulus intensity revealed that when subjects were not expecting the stimuli to vary, their changes in pain intensity ratings were significantly lower than when they were expecting the stimuli to vary (higher–middle: *non-matched* 1.1 ± 0.2; *matched* 2.1 ± 0.2, *T*(19) = 6.22, *p* = 0.000006) (Figure 2B). In other words, there was less increase in pain with the higher stimulus intensity when subjects expected no change in intensity. In contrast, there was no significant difference in pain intensity ratings during the lower stimulus intensity between the non-matched and matched expectation trials (middle–lower: *non-matched* 1.6 ± 0.2; *matched* 1.3 ± 0.2, *T*(19) = 1.26, *p* = 0.22). These pain intensity changes during matched versus non-matched trials were remarkably consistent; 19 of the 20 subjects rated the pain intensity as higher during the matched compared with the non-matched trials when the temperature was raised by 1°C (Figure 2C).

### Signal Intensity Changes

**FIGURE 3:**
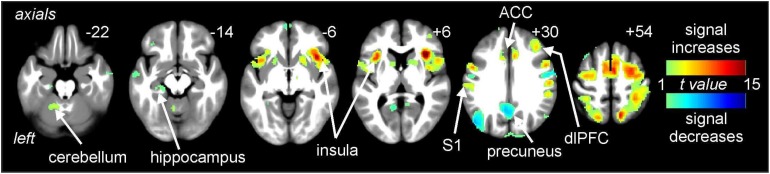
Signal intensity changes during painful thermal stimuli. Signal increases (hot color scale) occurred in a number of regions including the insula, primary somatosensory cortex (S1), anterior cingulate cortex (ACC), and dorsolateral prefrontal cortex (dlPFC). Signal decreases (cool color scale) occurred in areas such as the precuneus. Slice locations in Montreal Neurological Institute space are indicated at the top right of each image.

Noxious thermal stimuli (middle intensity, baseline scan) evoked signal intensity increases in numerous brain regions well known to be activated during pain. These regions included the cerebellar cortex, anterior insula, primary somatosensory cortex (S1), ACC, and dlPFC (Figure 3). Significant signal decreases occurred in the medial prefrontal cortex, precuneus and parietal association cortex.

**FIGURE 4:**
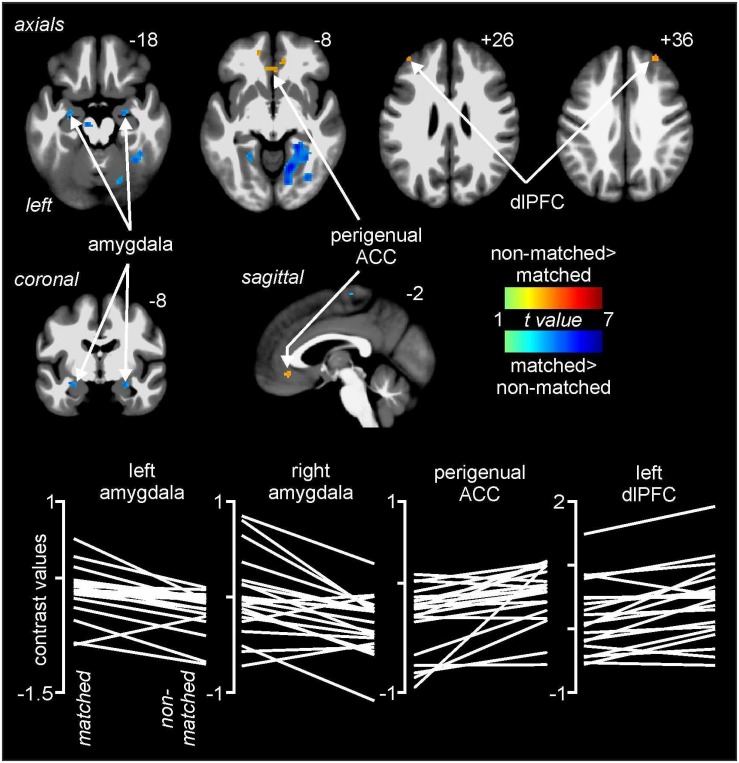
Differences in signal intensity changes with painful stimuli during non-matched compared with matched expectation scans using wholebrain images. Greater signal intensity increases (hot color scale) occurred during the non-matched compared with matched scans in the region of the perigenual anterior cingulate cortex (ACC) and the dorsolateral prefrontal cortex (dlPFC) bilaterally. Reduced signal intensity increases (cool color scale) occurred in the non-matched expectation scans in the amygdala bilaterally. Slice locations in Montreal Neurological Institute space are indicated at the top right of each image. The lower row are plots of contrast values for the left and right amygdala, perigenual ACC, and left dlPFC for each subject during matched and non-matched expectation trials.

Comparisons of signal intensity changes during non-matched and matched pain expectation scans revealed several regional differences. Greater signal intensity changes during matched versus non-matched pain scans occurred in the amygdala bilaterally (mean ± SEM contrast value: *left:* matched −0.08 ± 0.05, non-matched −0.37 ± 0.05; *right:* matched 0.03 ± 0.09, non-matched −0.29 ± 0.07), whereas reduced signal changes occurred in the area of the perigenual ACC (matched −0.20 ± 0.08, non-matched 0.05 ± 0.06) and left and right dlPFC (*left:* matched 0.05 ± 0.09, non-matched 0.32 ± 0.11; *right:* matched −0.10 ± 0.11, non-matched 0.25 ± 0.14) (Figure 4 and Table 1). Extraction of signal intensity changes revealed remarkably consistent patterns of signal change. Signal intensity changes were greater in the left amygdala (19 of the 20 subjects) and the right amygdala (16 of 20 subjects) during the matched versus the non-matched trials. Furthermore, in most subjects, there were greater signal intensity changes during the non-matched compared with the matched trials in the left dlPFC (17 of 20 subjects), the right dlPFC (16 of 20 subjects) and in the perigenual ACC (17 of 20 subjects) (Figure 4).

**TABLE 1:** Montreal Neurological Institute (MNI) coordinates, cluster size, and *t*-score for regions in which signal intensity increases or connectivity were significantly different during matched versus non-matched trials.

**Brain region**	**MNI Co-ordinates**			**Cluster size**	***t*-Score**
	***x***	***y***	***z***		

**Signal intensity change differences: matched versus non-matched**					
**Wholebrain analysis**					
Perigenual anterior cingulate cortex	4	32	−10	42	3.81
	−14	48		15	3.50
Left dorsolateral prefrontal cortex	−40	44	−8	10	3.91
Right dorsolateral prefrontal cortex	30	44	24	11	3.61
Parahippocampal gyrus	16	−62	36	400	5.43
Left amygdala	−30	−8	−8	15	4.14
Right amygdala	26	−8	−16	14	4.03
Brainstem specific analysis					
Right midbrain periaqueductal gray matter	4	−36	−5	5	3.35
Right substantia nigra	−10	−20	−15	30	5.55
Left substantia nigra	6	−20	−19	13	4.31

**Resting connectivity differences: matched versus non-matched**					

**Right amygdala**					
Primary somatosensory cortex	−62	−16	22	10	3.75
**Perigenual anterior cingulate cortex**					
Primary somatosensory cortex	−62	−12	24	15	3.67

**FIGURE 5:**
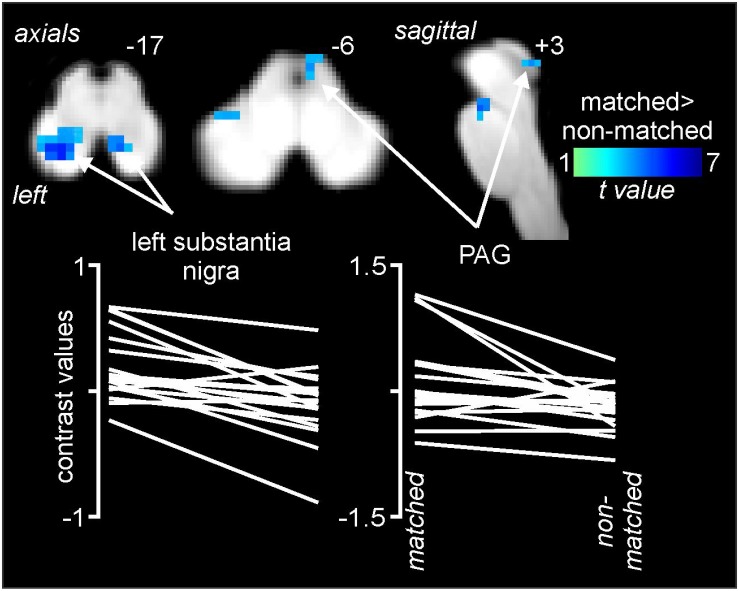
Differences in signal intensity changes during painful stimuli during non-matched compared with matched expectation scans for the brainstem-specific analysis. Reduced signal intensity changes (cool color scale) occurred during the non-matched compared with matched scans in the region of the substantia nigra bilaterally and the right midbrain periaqueductal gray matter (PAG). Slice locations in Montreal Neurological Institute space are indicated at the top right of each image. The lower row are plots of contrast values for the left substantia nigra and right PAG for each subject during matched and non-matched expectation trials.

Comparisons of signal intensity changes within the brainstem revealed three regions in which greater signal intensity changes occurred during matched versus non-matched pain scans (Figure 5 and Table 1). These encompassed the regions of the substantia nigra bilaterally (*left:* matched 0.18 ± 0.06, non-matched −0.10 ± 0.05; *right:* matched 0.25 ± 0.07, non-matched −0.04 ± 0.03) and the right midbrain PAG (matched 0.08 ± 0.11, non-matched −0.26 ± 0.06). Again, extraction of signal intensity changes revealed consistent patterns of greater signal change in the left substantia nigra (18 of 20 subjects), the right substantia nigra (16 of 20), and the right PAG (15 of 20) with matched relative to non-matched expectation trials.

### Psychophysiological Interactions Analysis Changes

**FIGURE 6:**
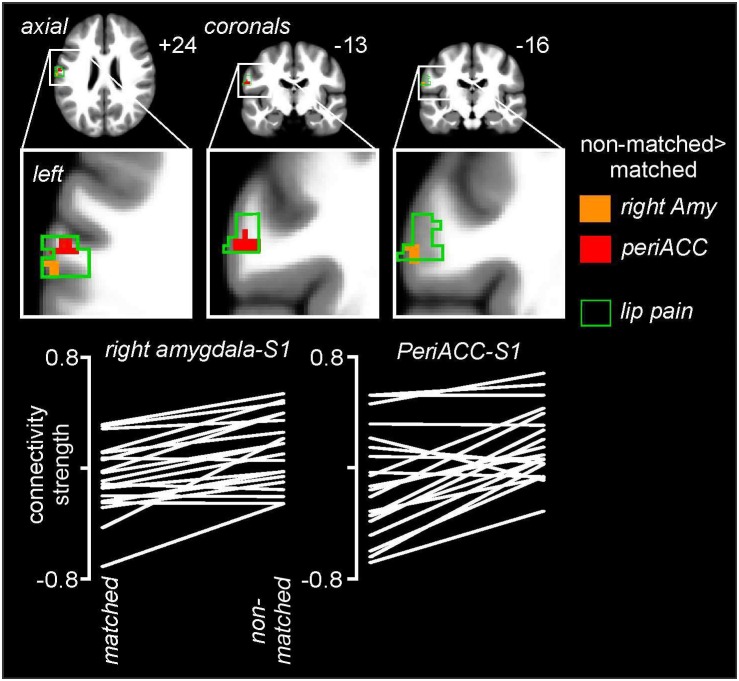
Noxious stimulus related connectivity differences during non-matched compared with matched expectation scans between the left primary somatosensory cortex (S1) and the right amygdala (Amy) and perigenual anterior congulate cortex (periACC) clusters. Greater S1 connectivity during non-matched trials occurred with both the right amygdala (orange shading) and perigenual ACC (red shading). The green outline indicates areas of the left S1 in which signal intensity increases during noxious thermal stimuli occurred during the baseline trial. Slice locations in Montreal Neurological Institute space are indicated at the top right of each image. The lower row are plots of connectivity strengths for the right amygdala-S1 and periACC-S1 for each subject during matched and non-matched expectation trials.

Psychophysiological interactions analysis revealed significant differences between matched and non-matched expectations in the noxious stimulus related connectivity between the perigenual ACC and right amygdala and the left primary somatosensory cortex (S1) (Figure 6 and Table 1). Connectivity between the right amygdala and S1 was significantly stronger during the non-matched compared to matched trials (mean ± SEM connectivity: matched −0.10 ± 0.06, non-matched 0.11 ± 0.05), as was that between the perigenual ACC and S1 (matched −0.10 ± 0.09, non-matched 0.16 ± 0.06). These differences were remarkably consistent; 16 of 20 subjects showed greater right amygdala-S1 connectivity strength and 17 of 20 subjects showed greater perigenual ACC-S1 connectivity strength during non-matched compared with matched trials. No significant S1 connectivity differences between the right or left dlPFC or left amygdala clusters occurred between matched and non-matched trials.

## Discussion

We found that an individual’s expectation can significantly alter both the perceived pain intensity and brain activation patterns during painful stimuli. We found differences in perceived pain intensity during varying noxious stimulus intensities when variations were expected, compared with when they were not. These differences were associated with altered activation in areas of the limbic system including the amygdala and the ACC, in addition to the dlPFC. We suggest that these activity pattern differences are related to the individual’s expectations not matching the stimulus presentation and may reflect the individual’s attempt to resolve this conflicting information.

When the individual expected the series of noxious stimuli to be of equal intensity but unbeknownst to them the stimulus intensity was increased, they rated the pain intensity lower than when they were expecting variations in stimulus intensity and the intensity was increased. That is, when a noxious stimulus intensity is unexpectedly increased, individuals do not rate the intensity as high as when an increase in pain is expected. Interestingly, the same did not occur when the pain intensity was lowered, with similar pain intensity ratings when expectations were non-matched and matched. Whilst we did not match stimulus presentations with sensory cues aimed at altering expectation, overall, the findings are consistent with previous stimulus expectation investigations. For example, Lorenz et al. (2005) matched auditory cues with low, moderate or strong noxious thermal stimulus intensities, and consistent with our results, when subjects expected a low intensity stimulus but received a high intensity stimulus, they reported significantly lower pain intensity than when the same high intensity stimulus was validly cued. Our results are consistent with this previous finding, although we found no significant difference when the stimulus intensity was lowered. Whilst our experimental paradigm was different in that there were no anticipatory cues, overall our results confirm that one can manipulate an individual’s subjective pain intensity ratings based on expectations. Furthermore, the evidence suggests that when expectation of higher stimulus intensity is not met, the resulting pain intensity is lower than when expectations are met, both in the presence and absence of anticipatory cues.

Noxious stimuli themselves activated a known circuitry in the brain; however, we found that even in known noxious processing pathways, brain activity patterns differed depending on whether the pattern of stimuli intensity matched the individual’s expectation. That is, we found multiple sites at which expectation-related and afferent-related information interact, which included the ACC, dlPFC, and amygdala. Previous studies have implicated the dorsolateral frontoparietal and limbic system in cue-based expectations (Corbetta and Shulman, 2002; Pessoa, 2009). Within the dlPFC, we found that signal intensity increased both when the stimuli matched expectations and when it did not, but increased less when the stimulus matched expectations. The dlPFC has been associated with numerous executive functions, including cognitive control, and has been consistently implicated in pain modulation and placebo (Wager et al., 2004). It has recently been shown that the dlPFC is involved in mediating analgesic effects such as conditioned pain modulation (Youssef et al., 2016). In placebo analgesia studies, increased dlPFC activity was associated with analgesia, and analgesic effects of perceived pain control were correlated with dlPFC activity (Wiech et al., 2006). It has been postulated that the prefrontal cortex “*represents the pivotal source of modulation that, at least within one conceivable pathway, initiates downstream analgesic activity and/or emotional modulation*” (Bingel and Tracey, 2008). Our data furthers this idea by showing that when an individual expects a stimulus that is not matched to the incoming sensory input, the dlPFC is recruited to a greater extent than when stimulus expectancy and sensory inputs are matched.

Whilst it has been proposed that the dlPFC alters pain perception via downstream inhibitory connections, it is possible that it also interacts with areas such as the ACC and amygdala to modulate pain perception. Indeed, tract tracing studies have revealed direct projections between the ACC and dlPFC (Selemon and Goldman-Rakic, 1988; Hoover and Vertes, 2007). We found that within the perigenual ACC, signal intensity decreased from baseline when the subsequent stimuli matched expectations, but increased when stimuli did not match expectations. It has been suggested that the integration of expectation with noxious afferent information is imperative for a complete cognitive experience of pain (Koyama et al., 2005) and it is possible that the dlPFC and ACC are critical for this process. The ACC is necessary for one to integrate error, conflict and reinforcement information and brain imaging studies have shown that the ACC is activated during conflicting stimuli or responses (Botvinick et al., 1999; Kerns et al., 2004). Additionally, the perigenual ACC is part of the “affective subdivision” of the cingulate cortex (Devinsky et al., 1995; Bush et al., 2000) with lesions to this region resulting in emotional lability (Hornak et al., 2003). This region also displays significant baseline hypoperfusion and hypometabolism in individuals with depression (Drevets et al., 1997; Mayberg et al., 1997). Whilst this raises the possibility that the altered ACC function found in this study is related to differences in emotional processing, given the lack of signal change during the matched expectation trial, we suggest that the difference in activation is more likely related to an individual processing the mismatch between sensory input and expectations.

In contrast to the ACC and dlPFC, we found that the amygdala displayed decreased signal intensity changes during non-matched expectations and almost no signal change from baseline when the stimuli intensity matched the individual’s expectations. More specifically, these changes were located primarily in the region encompassing the basal amygdala sub-nucleus. The lateral amygdala is the major input region, receiving inputs from sensory systems, including encoded pain; this region is widely considered to be the amygdala’s gatekeeper (LeDoux, 2007). This region sends information to multiple amygdala sub-nuclei including the basal nucleus which in turn projects to the striatum and cortex, including the ACC (Salzman and Fusi, 2010). Interestingly, a previous investigation linked altered dorsal amygdala activation to a verbal cue which provided expectation of a high or low intensity noxious stimulus (Atlas et al., 2010). Whilst we did not provide auditory cues, we found that when the individual’s expectation was not consistent with the incoming noxious stimulus intensity, amygdala activity was significantly reduced suggesting a critical role in processing stimulus expectation pain modulation. Indeed, Belova et al. (2007) measured amygdala neuronal responses to rewards and aversive air puffs when they were either expected or unexpected. They found that expectation often modulated responses to reinforcement, with enhanced responses occurring when reinforcement was not met with expectation. It is possible that these amygdala neurons participate in cognitive processes, possibly by feeding sensory information to areas such as the ACC and dlPFC so that the individual can respond and assess conflicting incoming noxious inputs with their immediate expectations. This mismatch could then result in a reduction in pain perception relative to a matched expectation-sensory input situation, possibly via projections to cortical areas processing noxious stimulus intensity. That we detected enhanced connectivity strength between the right amygdala and left S1 and the perigenual ACC and left S1 during the non-matched relative to matched trials, raises the possibility that these two regions modulate pain intensity by contacting S1.

In addition to altered activation and connectivity in higher brain regions, we also found differential activation of the region encompassing the midbrain PAG during matched compared with non-matched expectancy trials. The PAG is a key brainstem region involved in modulating incoming noxious inputs via projections to the rostral ventromedial medulla, a region which contains “ON” and “OFF” cells that can increase and decrease the excitability of neurons in the dorsal horn, respectively (Fields et al., 1983; Fields, 2004; Hellman and Mason, 2012; Salas et al., 2016). Whilst for the main part the PAG regulates this circuitry via an opiate-mediated mechanism, since opiate effects are generally prolonged and cue-based expectancy effects on pain must be transient and reversible, it has been suggested that opiates are unlikely to be instrumental in mediating stimulus expectancy effects on pain (Atlas and Wager, 2012). Alternatively, it has been suggested that cue-based expectancies may involve dopamine signaling, since phasic activity of midbrain dopamine neurons is considered to represent prediction error, that is, the difference between expected and actual stimuli reward (Sutton and Barto, 1981; Nasser et al., 2017). Indeed we also found altered activation in the region encompassing the substantia nigra and together with the PAG, this is consistent with the hypothesis that stimulus expectancy effects on pain may involve multiple neurotransmitter systems working in concert to alter pain processing both downstream at the level of the dorsal horn and upstream at the level of the S1 (Atlas and Wager, 2012).

### Limitations

There are a number of limitations of this investigation worth noting. Firstly, there may have been small differences in the movement required to shift the CoVAS rating system between the matched and non-matched expectation trials. However, we found no significant difference in the variability of any of the six movement directions between matched and non-matched trials and therefore we suggest that any differences in signal intensity or connectivity did not result from movement-related effects. Secondly, although we presented noxious stimuli within each fMRI series in random order, we were not able to counterbalance the presentation of the baseline, non-matched and matched scans themselves. This was necessary because the design required the individual to first experience a set of identical stimulus intensities from which they would form an expectation that the second scan would be identical to the first. It is possible that this sequence could have affected the results by producing, for instance, habituation. Nevertheless, we would argue that habituation did not occur since the mean pain intensity ratings during the three scans were not significantly different. Finally, we presented some of the fMRI results at an initial uncorrected statistical threshold and then performed cluster correction. Although such a procedure is often performed and our sample size of 20 subjects is not insignificant, a larger sample size would have likely allowed us to employ a more robust statistical threshold. To account for problems with cluster thresholding, e.g., poor spatial resolution for large clusters, we only considered cluster thresholding for clusters within meaningful anatomical areas. Therefore the limitation of cluster threshold, the difficulty with distinguishing between two conditions producing overlapping or distinct activation, was not applicable to our study. Another issue with cluster thresholding is the potential lack of clarity in visualization, so we ensured the boundaries of each cluster were clearly identified. Overall, since we found that at least 16 of the 20 subjects displayed the same pattern of response in the resulting significant clusters, we are confident that our results represent changes associated with the matching of stimulus intensity with individual expectations.

## Conclusion

Pain as an experience is influenced by various factors involving direct incoming sensory information along with cognitive, behavioral, personal, psychological, and social factors. One important psychosocial aspect includes expectation of the situation. In our study, we sought to explore whether expectation had effects on the intensity of the pain perceived. Our results supported our hypothesis that expectation has a significant influence on one’s perception of pain. Specifically, increases in stimulus intensities were perceived to be lower when they were increased above expectations whereas the magnitude of perceived pain evoked by the same stimuli was higher when variations in intensity were expected. Our fMRI results demonstrate differences in brain activation patterns when expectations met variations in stimuli intensities versus when expectations were not met, with effects seen in areas such as the amygdala, ACC and dlPFC. We conclude that an individual’s subjective perception of pain intensity can be manipulated based on their expectations. It would be interesting for future studies to investigate whether these theories could be applied clinically, for example, whether managing a patient’s expectations in a dental or surgical setting can have a positive influence on their pain experience. This is a worthwhile area to explore as dental and medical phobias are a major barrier to healthcare delivery. The ability to improve a patient’s pain experience would be greatly beneficial to clinicians and patients during the treatment phase and may help reduce dental and medical anxieties in our patients.

## Data Availability Statement

The raw, anonymized data supporting the conclusions of this article will be made available by the authors, without undue reservation, to any qualified researcher.

## Ethics Statement

The studies involving human participants were reviewed and approved by HREC, The University of Sydney. The patients/participants provided their written informed consent to participate in this study.

## Author Contributions

LH designed the study, led analysis and interpretation of the data, and wrote the manuscript. FD, AY, SL, ST, RA, EM, GM, and CP helped significantly with data collection and analysis, and editing of the manuscript. PM helped with analysis and editing of the manuscript.

## Conflict of Interest

The authors declare that the research was conducted in the absence of any commercial or financial relationships that could be construed as a potential conflict of interest.
